# The 3′ region of the ZPA regulatory sequence (ZRS) is required for activity and contains a critical E-box

**DOI:** 10.3389/fcell.2025.1569573

**Published:** 2025-07-02

**Authors:** Kathryn F. Ball, Stephen Manu, Abbie K. Underhill, Jeanyoung Kim, Jessica C. Britton, Sarah R. Rudd, Madison M. Malone, Japhet Amoah, Allen Cooper, Charmaine Pira, Kerby C. Oberg

**Affiliations:** ^1^ Pathology and Human Anatomy Department, Loma Linda University, Loma Linda, CA, United States; ^2^ Basic Sciences Department, Loma Linda University, Loma Linda, CA, United States

**Keywords:** limb development, sonic hedgehog, cis-regulatory module, enhancer, Hand2, HoxD13

## Abstract

**Background:**

During development, Hand2 and Hoxd13 transcription factors (TFs) regulate Sonic hedgehog (Shh) expression in the zone of polarizing activity (ZPA) in the distal posterior limb mesoderm. The ZPA regulatory sequence (ZRS) is a conserved, limb-specific enhancer that controls Shh expression. The ZRS can be divided into 5′, central, and 3′ subdomains, each with an E-box site that can bind basic helix-loop-helix (bHLH) TFs like Hand2. In addition, two Hoxd13 sites are present in the 5′ and central subdomains. Hand2 purportedly binds the ZRS through the central E-box, and both Hand2 and Hoxd13 have been shown to activate the ZRS *in vitro*. We hypothesized that the central E-box was required for activity, while the other E-boxes and Hoxd13 sites localize ZRS activity to the distal posterior limb mesoderm.

**Methods:**

To identify the functional role of each subdomain, we generated three ZRS fragments (5′, central, and 3′) and combined fragment constructs to test subdomain collective contributions. Additionally, we disrupted the five binding sites, alone or in concert, using site-directed mutagenesis. All ZRS constructs were cloned into a GFP reporter and evaluated in an *in vivo* chicken limb bioassay. We validated our findings using select ZRS constructs in transgenic mice.

**Results:**

We found that the 3′ fragment was necessary for ZRS activity, while the 5′ and central fragments had no activity alone or when combined. However, combining the 3′ fragment with the 5′ fragment restored robust activity. Further, mutation of all five binding sites markedly reduced ZRS activity. Reinstating each of the Hoxd13 sites restored focal activity, while restoring the 5′ and central E-boxes had little effect. However, the 3′ E-box proved sufficient for robust activity even in the absence of the other four binding sites.

**Conclusion:**

Our data indicate that the ZRS 3′, not the central, subdomain is necessary for activity and contains the 3′ E-box that Hand2 likely uses to induce Shh expression, while the 5′ and central E-boxes appear to be inhibitory. Our data also suggest that the Hoxd13 binding sites promote localized activity within the ZPA.

## 1 Introduction

Sonic hedgehog (Shh) is a secreted signaling factor that directs morphogenesis in several organs during development including the neural tube, early gut, and limb. The zone of polarizing activity (ZPA) refers to a small subpopulation of mesenchymal cells in the posterior distal aspect of the developing limb that secrete Shh to direct anterior-posterior (AP) patterning. Shh knock-out (KO) in mice results in loss of posterior limb structures such as the ulna and fibula in the zeugopod, and all but a single digit in the autopod (Chiang et al., 2001; Kraus et al., 2001). Despite Shh’s pivotal role in limb patterning, the mechanisms that maintain its expression within the ZPA during progressive limb outgrowth remain unclear.

**FIGURE 1:**
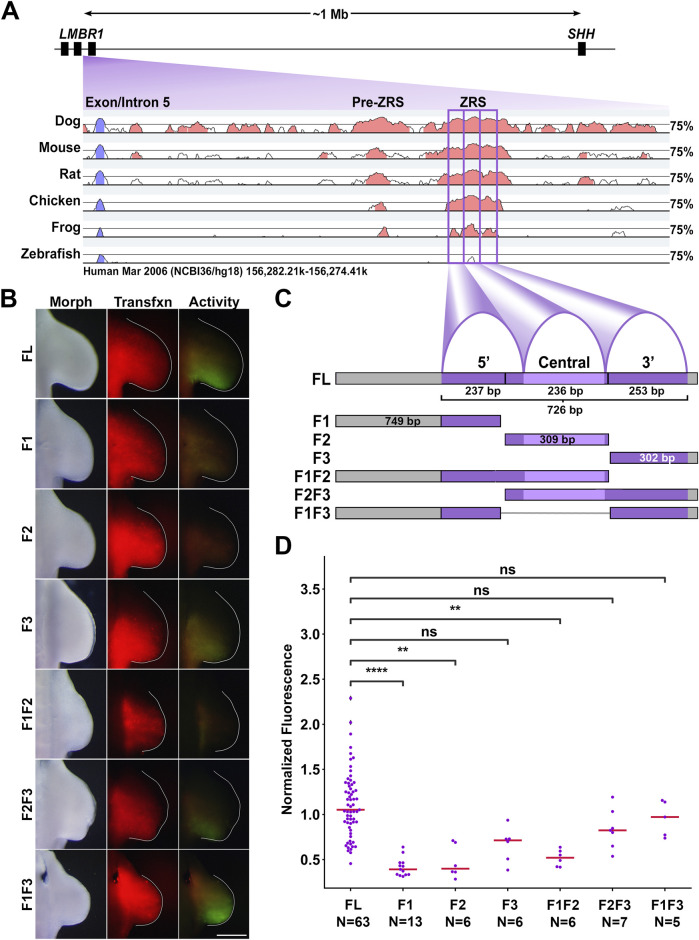
The 3′ subdomain of the ZRS is required for activity. **(A)** Diagram of the ZRS locus relative to Shh and pairwise conservation of each listed species in comparison to the human sequence (VISTA point). The ZRS subdomains are boxed in purple. **(B)** Activity of ZRS and subdomain fragments in chicken forelimbs. Morph: morphology, Tfxn: transfection control. Images are dorsal view with top: anterior, right: distal. Scale Bar = 1 mM. **(C)** Diagram of the conserved chicken ZRS, peaks, and Erase-a-base fragments used in this study. Further details on the sizes and composition of the individual and combined fragments are present in the methods and Supplementary Figure S5 and Supplementary Tables S2, S5–7. **(D)** Swarm plots of fragment activity (GFP intensity) normalized to transfection control (RFP intensity). Changes in activity were compared with a Kruskal–Wallis test followed by Dunn’s test. ** = p < 0.01, **** = p < 0.0001, ns = not significant. N refers to the number of embryos per group. Experimental groups were repeated in at least three independent experiments.

*Cis*-regulatory modules (CRMs) are DNA sequences that sense cellular cues for tissue-specific transcription factors (TFs). These sequences, when in context of their chromatin environment and topologically associated regulatory domains (TADs), regulate associated target genes. The ZPA regulatory sequence (ZRS) is a limb-specific CRM located approximately one million bases upstream of the *Shh* promoter within intron five of the *Lmbr1* gene. The ZRS is necessary for Shh expression in the ZPA, as demonstrated by the loss of Shh expression after a spontaneous ZRS microdeletion in chickens (Ros et al., 2003) or after ZRS KO in mice (Sagai et al., 2005). The ZRS can be divided into three subdomains that are conserved across vertebrate species, hereafter called: 5′, central, and 3’ (Figure 1A, purple boxes). Investigating ZRS architecture can help identify themes in CRM function and elucidate the overarching principles of regulatory DNA.

The Hoxd13 and Hand2 TFs regulate Shh expression in the limb. Hoxd13 contributes to AP polarity, and early anterior Hoxd13 misexpression results in anterior Shh expression (Zakany et al., 2004). Hand2 is necessary for Shh expression; Hand2-deficient mouse limb buds display a phenotype similar to Shh loss-of-function limbs (Galli et al., 2010). Conversely, anterior Hand2 misexpression in the limb bud produces ectopic Shh expression leading to mirror-image digit duplication (Charite et al., 2000). Hoxd13 and Hand2 bind the ZRS and each other; they can also independently transactivate ZRS-luciferase *in vitro* and, when combined, can transactivate ZRS synergistically (Capellini et al., 2006; Galli et al., 2010).

Hand2 is a basic helix-loop-helix (bHLH) TF that forms homo- and heterodimers with other bHLH factors such as Hand1, Twist1, E12, and E47 (Dai and Cserjesi, 2002; Firulli et al., 2005; Koyano-Nakagawa et al., 2022). Two bHLH monomers must dimerize to form a functional TF, and since each monomer contributes its DNA binding domain to make half of the whole DNA binding region, dimer composition can affect the affinity to a binding site (Dai et al., 2002; Firulli et al., 2007). Even small changes in binding affinity can result in a pathological phenotype (Lim et al., 2024).

TFs in the bHLH family bind E-boxes, hexamers with a core “CANNTG” motif. An E-box with Hand2’s consensus binding sequence (CAGATG) in the central ZRS subdomain is purported to be the Hand2 binding site (Galli et al., 2010; Osterwalder et al., 2014). Other factors including Snail and Slug, zinc-finger TFs, and Hey1 are expressed in the early limb and could also bind this E-box. We set out to interrogate this E-box along with two others within the highly conserved ZRS to determine their relevance to ZRS activity.

Efforts have been made to map the ZRS; however, this work is incomplete. Characterizing the ZRS TF binding site (TFBS) landscape is critical to both understanding development and clinical Shh dysregulation. More than 30 single-nucleotide variations (SNVs) within the ZRS have been documented, most of which result in preaxial polydactyly (PPD) and/or triphalangeal thumb (TPT) (Supplementary Table S1). A majority of the human SNVs (22/34) are located within the central ZRS subdomain suggesting this region is susceptible to perturbation (Supplementary Figure S1). In this study, we used isolated ZRS sequences either in transient episomal (chicken) and randomly integrated (murine) reporter vectors to evaluate the intrinsic functional domains of the ZRS. We demonstrate the 3′ E-box is critical for ZRS activation, the 5′ and central E-boxes are repressive, and the Hox sites are activating.

## 2 Materials and methods

### 2.1 Plasmid construction

To test ZRS activity *in ovo*, we generated episomal expression constructs with ptk-EGFP plasmid (a gift from Dr. Masanori Uchikawa, Osaka University, Japan) (Uchikawa et al., 2003), which contains the minimal HSV TK promoter linked to an enhanced GFP reporter gene. Chicken ZRS (cZRS, a 1,373 bp fragment, Assembly IDs UCSC: GRCg6a/galGal6 and NCBI: 1668981, Chr2:8,553,160-8,554,532) or human ZRS (hZRS, a 1,198bp fragment, Assembly IDS UCSC Hg38 and NCBI: GRCh38.p14, Chr7:156,791,072-156,792,269) was isolated by PCR from genomic DNA and ligated into pTK-EGFP at the XhoI restriction site. Constructs containing individual conserved peaks were generated through progressive digestion with the Erase-a-Base system (Promega, Madison, WI). The following constructs were generated (full sequences found in Supplementary Table S2):

F1 (749 bp total): 168 bp of the 5′ subdomain +581 bp of the adjacent upstream DNA).
F2 (309 bp total): 236 bp of the central subdomain +60 bp of the adjacent 5′ subdomain and 11 bp of the adjacent 3′ subdomain.
F3 (302 bp): 236 bp of the 3′ subdomain +66 bp of the adjacent 3′ DNA.
F1F2 (1065 bp): F1 (749 bp) + 236 bp of the central subdomain and 11bp of the adjacent 3′ subdomain.
F2F3 (615 bp): 236 bp of the central subdomain and 253 bp from the 3′ subdomain +60 bp from the adjacent 5′ subdomain and 66 bp of the adjacent 3′ DNA
F1F3 (1058 bp): 175 bp of the 5′ subdomain (and 581 bp of the adjacent upstream DNA) + F3 (302 bp)

The pCAGGS-RFP plasmid (a gift from Dr. Cheryl Tickle, University of Dundee, Scotland) (Das et al., 2006) was co-electroporated to verify transfection. Plasmids were isolated and purified using the EndoFree Plasmid Maxiprep Kit (Qiagen, Valencia, CA).

### 2.2 Site-directed mutagenesis

To disrupt transcription factor binding, we altered three-to-four core bases of each putative binding site with the QuikChange Multi Site-Directed Mutagenesis Kit (Agilent Technologies, Santa Clara, CA) while also introducing a restriction site for screening. Mutant sequences were analyzed with CiiiDER (Gearing et al., 2019) to ensure no new binding sites relevant to limb development were introduced. NEB5-α competent cells were transformed with mutated constructs. Transformants were screened using the new restriction site and constructs were confirmed via Sanger sequencing (Eton Bio, San Diego, CA). All genomic and mutagenic primers are listed in Supplementary Table S3.

### 2.3 Targeted regional electroporation (TREP)

Chicken embryos were staged according to the Hamburger and Hamilton (HH) method (Hamburger and Hamilton, 1951). The embryonic coelom within the lateral plate mesoderm of stage HH14 embryos was injected with DNA solution (2 μg/μL pTK-ZRS-EGFP, 0.2 μg/μL pCAGGS-RFP with Fastgreen and Tris-EDTA buffer). Plasmids were electroporated into the presumptive forelimb using the CUY-21 Electroporator (Protech International Inc., Boerne, TX) as previously described (PIRA et al., 2008). Embryos were incubated for 48 h post-electroporation then harvested. We visualized fluorescence with a Leica MZ FLIII fluorescence stereo microscope using 41012 HQ:FLP FITC/EGFP and 10446365 TXR filters (Chroma Technology Corp., Brattleboro, VT); images were captured with a Sony DKC-5000 camera and acquired using *Adobe Photoshop* (version 6.0). The inclusion criteria for the chicken embryo limbs can be found in Supplementary Table S4.

### 2.4 Image analysis

Image analysis was performed using a workflow written in *Python* (3.9.12). In short, images were converted to grayscale, passed through a bilateral denoise filter, then the region of ‘Limb’ was determined using a combination of Otsu thresholding and manual input (to separate limb from body wall) on the light image. To limit RFP measurement to relevant tissue only, three different masks were made for each limb: the Limb mask excluded background and non-limb tissue, the Posterior mask excluded tissue that might be transfected, but would not express wild-type activity, and the ZPA mask that limits measurement to the region of active *Shh* transcription. Diagrams of the masks can be seen in Figure 3C. We used the Posterior mask for all image analyses in this paper except for the Hoxd13 mutant analysis shown in Figure 4C. The region of transfection was determined using the masked RFP image and Otsu thresholding. The region of enhancer activity was determined by applying the ‘RFP’ mask to the GFP image combined with Otsu thresholding. Pixel number and intensity were measured within the appropriate mask (RFP on the RFP image, GFP on the GFP image), and relative enhancer activity was determined by normalizing total GFP intensity to total RFP intensity. This normalization accounts for differences in transfection. A *Jupyter* notebook of the code used is available at https://github.com/KateBall/Quantitative_Image_Analysis under the GNU Public License (GPL, ver. 3). A preprint describing the method in detail can be found at (Ball et al., 2025).

### 2.5 Binding site affinity analysis

Relative binding affinity was calculated using protein binding microarray (PBM) data from the UniProbe database (http://thebrain.bwh.harvard.edu/uniprobe/index.php) (Hume et al., 2015) and processed using *Python* code adapted from (Lim et al., 2024). The adapted code is available at (https://github.com/KateBall/ZRS-2025). Human sequence was used for our queries, the PBM dataset was generated with mouse TF. For each transcription factor, the relative affinity values represent a ratio of the median intensities of the given 8mer over the factor’s optimal 8mer from the PBM data.

### 2.6 Multiple alignment using fast fourier transform (MAFFT) analysis

Multiple alignment using fast Fourier transform (MAFFT) of the conserved ZRS regions for human, dog, mouse, rat, chicken, frog, and zebrafish was performed using the web browser form of MAFFT available through EMBL-EBI to evaluate conservation of transcription factor binding sites of interest. Applied output parameters: gap open penalty: 1.53; gap extension penalty: 0.123; tree rebuilding number: 2; max iterate: 2; FFTS: none. Specific assemblies and coordinates used to isolate sequences used in MAFFT are listed in Supplementary Table S5.

### 2.7 Transgenic mice

Human ZRS (hZRS) and its Δ5 mutant (hZRSΔ5) were cloned into the HSP68-LacZ plasmid kindly provided by Dr. Nadav Ahituv, UC San Francisco, CA (Pennacchio et al., 2006). The constructs were used to generate transgenic mouse embryos via random integration (Cyagen transgenic service, Santa Clara, CA). Embryos were harvested at e12.5 and processed for detection of LacZ activity.

### 2.8 Statistical analysis

TREP data were collected over at least three separate experiments per group. Individual data points, each corresponding to the forelimb of a separate embryo, are shown via swarm plot; medians are in red. The reported sample sizes (N) on each plot correspond to the number of embryos in each group. Note that the same data for chicken wild-type ZRS (referred to as “FL” and “WT”) are shown in both Figures 1, 3. Quantitative data were analyzed with the *Python* modules *Pandas*, *NumPy*, *SciPy*, and *scikit_posthocs*, and were visualized with *Matplotlib* and *Seaborn*. To check for normality, we visually inspected the data using histograms and used the Shapiro-Wilk test for normality. Outliers were identified using the interquartile range method, but not dropped as it would necessitate removing some of the smaller groups from analysis. Statistically significant (p < 0.05) differences were determined with the Kruskal–Wallis test followed by Dunn’s multiple comparisons test with Bonferroni p-value correction or the Mann-Whitney U test when only two groups were compared. * = p < 0.05; ** = p < 0.005; *** = p < 0.0005; **** = p < 0.0001. A *Jupyter* notebook of the complete statistical analysis can be found at (https://github.com/KateBall/ZRS-2025).

Note: All figures were made using *Adobe Photoshop (CC)*. Subfigures containing plots, masks, or limbs with contours also used *Matplotlib 3.5.1*, *Seaborn 0.12.2*, and *Statannotations 0.4.4*. Original psd files are available upon request. Any alterations made and are intended for clarity and aesthetic purposes only. All quantitative data used in this study are collected from raw, unaltered image files.

## 3 Results

### 3.1 The ZRS 3′ region is required for activity

To uncover the regulatory role of the ZRS subdomains, we inserted an isolated fragment of the chicken ZRS (1373 bp fragment including adjacent DNA, 581 bp 5′ and 66 bp 3′) into a GFP reporter construct. Transfection of this full length (FL) reporter into presumptive upper limbs of embryonic chickens demonstrated activity that overlapped the ZPA after 48 h s of incubation (Figure 1). We then generated fragments of the ZRS using the Erase-a-base system (Promega). The 5’ (F1) fragment (749 bp) contained 168 bp of the 5′ subdomain and the 581 bp of adjacent 5′ DNA. The central (F2) fragment (309 bp) contained 236 bp of the central subdomain plus 60 bp of the 5’subdomain and 13 bp of the 3′ subdomain. The 3’ (F3) fragment (302 bp) contained 236 bp of the 3′ subdomain and 66 bp of adjacent 3′ DNA. The 3′ fragment (F3) demonstrated activity, though less intense, was not significantly different from full-length (FL) wild type (p > 0.05); while the 5′ and central fragments (F1 and F2, respectively) had significantly less activity compared to FL (p < 0.0001 and p < 0.01 respectively). The lack of activity in the central fragment was surprising because Hand2 reportedly binds the central E-box (E-box 2) (Osterwalder et al., 2014). This shifted our attention to the 3′ fragment since it retained activity consistent with a critical role in ZRS activation.

The combination of the 3′ fragment with the 5′ fragment (F1F3) exhibited more intense activity than the 3′ fragment alone (Mann-Whitney U Test p = 0.017) and was similar to wild type in both pattern and intensity. The construct combining the 5′ and central portions of the ZRS (F1F2) still lacked statistically significant activity when compared to the wild type ZRS. We also tested the F2F3 fragment, and did not find a significant increase over F3. These data indicate that the 5’ (F1) subdomain contributes to activity, but only in the presence of the 3′ region (F3).

### 3.2 Loss of Hoxd13 binding sites 1 & 2 and E-boxes 1-3 nearly abolishes ZRS activity

**FIGURE 2:**
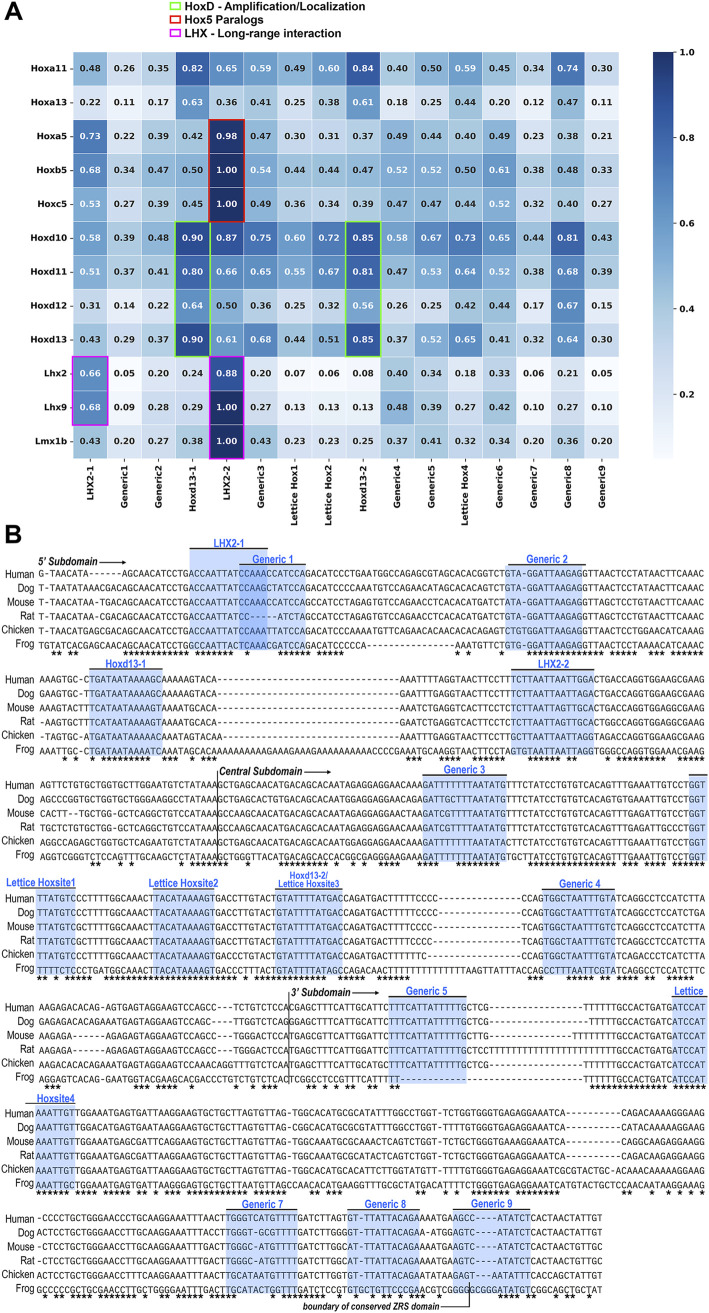
Hox Site Affinity. **(A)** A heatmap of the relative *in vitro* binding affinity of several homeodomain transcription factors (TFs) (y-axis) to each possible homeobox sequence in the human ZRS (x-axis), as measured by protein binding microarray (PBM) using mouse TF. Each TF is tested against a microarray of all possible DNA 8mers (4^8^ = 65,536 8mers). Relative affinity for each transcription factor-binding site pair is the median intensity of the binding site 8mer over the intensity of the transcription factor’s optimal 8mer (maximum intensity of all possible 8mers) and is given as a ratio on a scale of 0 (white) to 1(dark blue). The 8mer with the highest intensity level for a given transcription factor is set equal to 1. The green boxed cells indicate the preferred sites (highest binding affinity) of the 5′ Hoxd transcription factors (Hoxd10-13) associated with ZRS activation, The red boxed cells indicate the preferred binding site for the Hox5 paralogs, which have been associated with anterior ZRS inhibition, although this site is in the 5′ subdomain, not the expected Hoxd13-2 site within the central subdomain. The pink boxed cells identify the preferred binding sites of the LIM domain transcription factors (Lhx2, Lhx9 and Lmx1b) associated with long-range enhancer activation. **(B)** MAAFT alignment of full ZRS sequence from human to frog, with conserved base pairs indicated with an asterisk below (Madeira et al., 2024). Binding sites evaluated by PBM in **(A)** are annotated. Note the high degree of conservation of the evaluated binding sites. Generic 5, though absent in frog, is fully conserved otherwise. Binding site “Generic 9” exists beyond the boundary of the region we have isolated as the conserved 3′ subdomain, and the endpoint of the 3′ subdomain has been noted accordingly.

**FIGURE 3:**
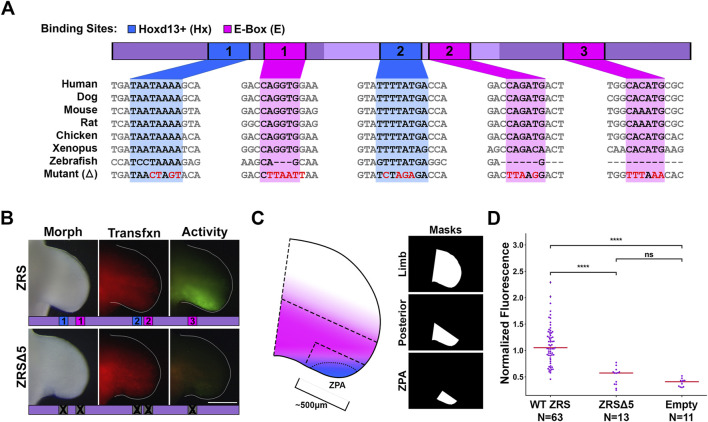
Loss of Hoxd13 binding sites and E-boxes reduces ZRS activity. **(A)** Diagram of key transcription factors and binding sites in this study. Asterisk indicates reported Hand2 binding site. **(B)** Activity of wild type ZRS (WT) or with five binding sites mutated (ZRSΔ5). **(C)** Diagram showing Hand2 (pink) and Hoxd13 (blue) expression pattern overlap. Masks indicate the three regions in which fluorescence was measured and correspond to dashed regions on the forelimb diagram. **(D)** Swarm plots of reporter activity (GFP intensity) normalized to transfection control (RFP intensity). Data collected using the Posterior mask. Changes in activity were compared with a Kruskal–Wallis test followed by Dunn’s test. **** = p < 0.0001, ns = not significant. N refers to the number of embryos per group. Experimental groups were repeated in at least three independent experiments.

Since Hand2 and Hoxd13 have been shown to work together to activate the ZRS *in vitro* (Galli et al., 2010), we set out to identify probable binding sites for each. Hox TFs are known for their promiscuity, in binding to a variety of Hox binding sites, making their orchestration in development complex to disentangle. To determine the most likely location(s) of Hoxd13 interaction within the ZRS and the interplay with other Hox factors, we evaluated the potential Hox binding sites within the ZRS. We compared the relative affinities of all homeobox sites within the conserved ZRS to homeodomain TFs known to be expressed in the limb using protein binding microarray (PBM) data (Figure 2). Hoxd13 has a higher relative affinity for the two sites targeted in this study with C/TAATAAAA motifs (Hoxd13 sites 1 and 2) than for any of the other potential Hox binding sites. Some of the other 5′ Hox TFs also favor these sites (green boxed cells), suggesting these sites may provide a competitive mechanism to activate and localize ZRS activity during limb development. Unfortunately, PBM data was not available for key bHLH factors such as Hand2, so an equivalent E-box analysis could not be performed. Thus, we evaluated three E-boxes within the ZRS that are conserved across divergent species (Figure 3A).

E-boxes 1, 2, and 3 are in the 5′, central, and 3′ subdomains, respectively. E-box 2 (CAGATG) in the central subdomain is Hand2’s predicted binding motif (for more detail see Supplementary Figure S2). We mutated all five of these binding sites in concert (ZRSΔ5) as a screening process to see if any of the sites had functional relevance and found that loss of all five binding sites nearly abolished ZRS activity (Figure 3).

### 3.3 The presence of at least one functional Hoxd13 binding site in the ZRSΔ5 restores focal activity

**FIGURE 4:**
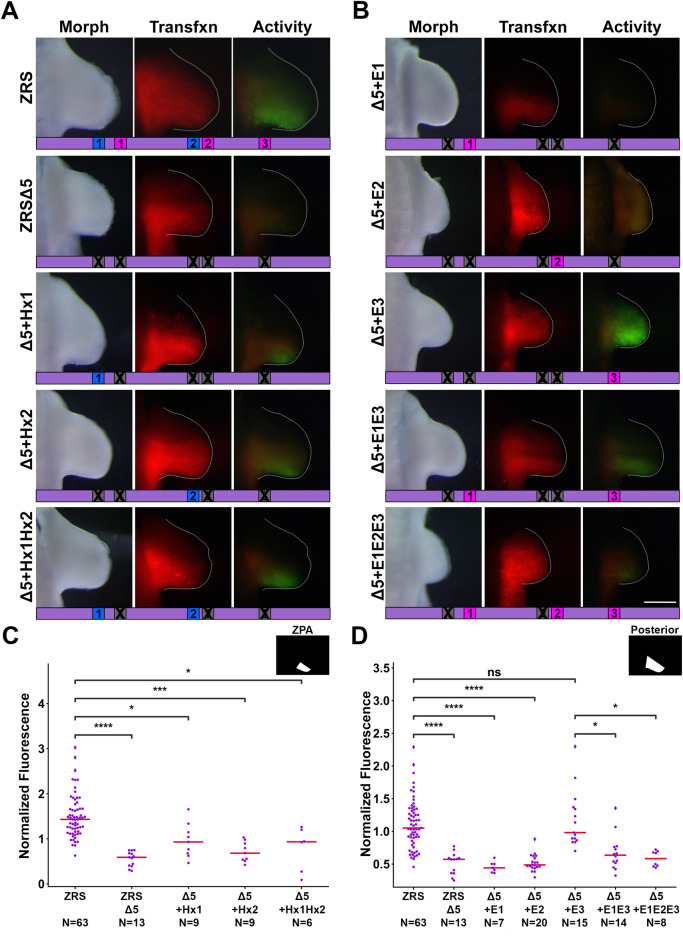
E-box and Hoxd13 binding sites differentially regulate ZRS activity. **(A)** Activity of ZRS: wild type (WT) or with five binding sites mutated (ZRSΔ5), restoration of each Hoxd13 site, alone and in concert. **(B)** Restoration of each E-box, alone and in concert. Diagrams below each forelimb image show which binding sites are present or absent in the given construct. **(C, D)** Swarm plots of reporter activity (GFP intensity) normalized to transfection control (RFP intensity). Activity was measured using the ZPA mask in **(C)** and the Posterior mask in **(D)**. Changes in activity were compared with a Kruskal–Wallis test followed by Dunn’s test. * = p < 0.05, *** = p < 0.001, **** = p < 0.0001, ns = not significant. N refers to the number of embryos per group. Experimental groups were repeated in at least three independent experiments.

To determine the relative contribution of each binding site to ZRS activity, we used the ZRSΔ5 as a baseline and restored each binding site individually and with the others of its class. With this assay, the signal measured is the result of accumulated GFP within cells having a history of ZRS activation over the 48-h incubation period. WT activity is the result of early ZRS induction, presumably from Hand2, which is expressed prior to limb outgrowth, and maintained by Hoxd13 a few stages later in the limb bud. The presence of at least one Hoxd13 binding site (ZRSΔ5+Hx1, ZRSΔ5+Hx2, or ZRSΔ5+Hx1Hx2), produced activity that appears more focal than wild type (Figure 4). To capture the differential activity in the ZPA domain, we used a ZPA mask to quantitate fluorescence (Figure 3C). We found that within the ZPA domain, ZRSΔ5+Hx1 recovered activity with intensity that was not significantly different from wild type, though ZRSΔ5+Hx2 was less (Figure 4C). Thus, the focal activity of the ZRSΔ5+Hx1 and ZRSΔ5+Hx2 constructs may reflect a late maintenance-related activation with reduced GFP accumulation. Interestingly, F1F2 has no activity despite containing both Hoxd13 binding sites, suggesting that other binding sites in the 3′ region are necessary to support Hoxd13–related activation.

### 3.4 E-box 3, and not the canonical Hand2 binding site, restores ZRS activity

In our experiments, restoring E-box 1 or E-box 2 in ZRSΔ5 did not significantly increase activity (Figures 4B,D). However, restoring E-box 3 in the context of ZRSΔ5 produced activity significantly greater than wild type, suggesting the 3′ E-box drives ZRS activity and may be the site Hand2 uses to activate the ZRS. Surprisingly, ZRS activity in the presence of the 3′ E-box 3 in combination with E-boxes 1 (ZRSΔ5+E1E3) or E-boxes 1 and 2 (ZRSΔ5+E1E2E3), results in a reduction of activity (Figure 4D), indicating E-boxes 1 and 2 perform an inhibitory role.

E-box 3 restores ZRS activity despite the absence of two Hoxd13 sites. This may be possible because other Hox binding sites are present in the ZRS and Hox TFs are known to be promiscuous. Thus, it is possible that Hoxd13 is acting on ZRS through other Hox binding sites. We initially suspected that loss of the purported Hand2 binding site, E-box 2, would be sufficient to eliminate ZRS activity. However, others have shown that the central ZRS subdomain (F2) is not essential for activity although it is important for regulating the level and location of transcription (Lettice et al., 2017). We also found that the ZRS maintained activity following site-directed mutagenesis of the central E-box alone, consistent with Lettice and colleagues (Supplementary Figure S3).

### 3.5 The conserved Hoxd13 and E-Box sites are also critical for human ZRS activity

**FIGURE 5:**
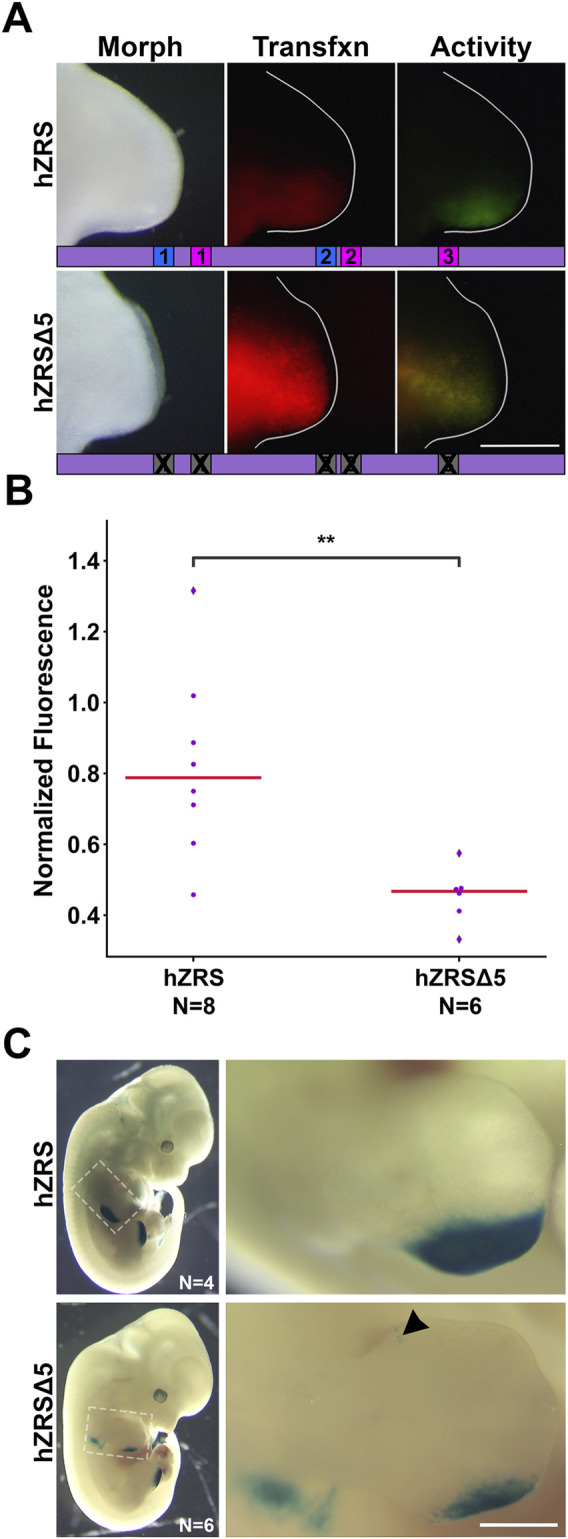
The importance of the five binding sites is conserved in human ZRS. **(A)** Activity of wild type human ZRS (hZRS) or with five binding sites mutated (hZRSΔ5) in the chicken forelimb. **(B)** Swarm plots of reporter activity (GFP intensity) normalized to transfection control (RFP intensity). Changes in activity were compared with a Mann-Whitney U test. ** = p < 0.01. N refers to the number of embryos per group. Experimental groups were repeated in two-three independent experiments. **(C)** hZRS and hZRSΔ5 activity in mouse embryos harvested at e12.5. Arrowhead points to ectopic anterior activity. Size bar: 1 mm.

To determine whether the necessity of the five binding sites is conserved across species, we repeated the *in vivo* bioassays using human ZRS (hZRS) and found that the absence of the five binding sites (hZRSΔ5) also depleted hZRS activity (Figures 5A,B). We then interrogated hZRS in the transgenic murine model using a β -galactosidase assay that results in blue precipitate in locations that have had ZRS activity. Although both wild type and hZRSΔ5 showed evidence of ZRS activity (Figure 5C), the wild type hZRS had blue precipitate encompassing digital rays 4 and 5. However, hZRSΔ5 activity was substantially reduced and restricted to the distal tips of digital rays 4 and 5, indicating the five binding sites are needed for normal ZRS activity. The hZRSΔ5 construct resulted in some activity (arrowhead) outside of the ZPA-related region stained by the wild type ZRS in some embryos, despite verifying that no new binding sites were introduced. The additional ectopic activity may reflect the difference in insertion sites. Supporting this perspective, the embryos with ectopic staining also had a similar pattern of increased global activity. Interestingly, one embryo with the wild-type construct also produced some anterior ectopic staining (Supplementary Figure S4) and correspondingly had a mild increase in global activity.

## 4 Discussion

The ZRS is an ∼800 bp highly conserved cis-regulatory module found within intron five of the *Lmbr1* gene (Lettice et al., 2003). Previous work has identified several important features of the ZRS including ETS and Hox binding sites that can be described as molecular rheostats controlling the relative level of ZRS activity (Lettice et al., 2012; 2017; Lim et al., 2024). The ZRS can be divided into three subdomains (5′, central, and 3′) based on the degree of sequence conservation from human to frog (Figure 1). Our study also uncovered transcription factor binding sites that are critical for ZRS activity. Each of the three subdomains of the ZRS contain an E-box with the capacity to interact with basic helix-loop-helix transcription factors such as Hand2. We also evaluated two Hoxd13 binding sites, one in the 5′ subdomain and one in the central subdomain.

Disruption of the three E-boxes and two Hoxd13 binding sites (ZRSΔ5) nearly abates ZRS activity (Figures 3, 4). By restoring each of the sites individually and in combination, we discovered that the 3′ E-box (E-box 3) and both Hoxd13 sites (1 and 2) can activate transcription, while the 5′ and central E-boxes (E-boxes 1 and 2) play inhibitory roles. Thus, we conclude that E-box 3 is the most likely site for Hand2 interaction.

**FIGURE 6:**
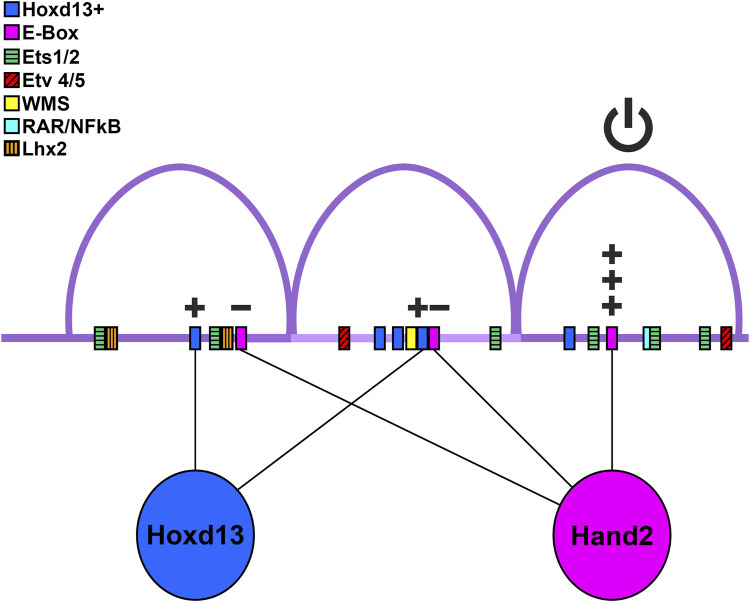
Model of binding site utilization. Diagram of ZRS with possible transcription factor-binding site interactions. Purple arcs are the three conserved peaks. Hoxd13 and its putative binding sites are shown in blue, Hand2 and E-boxes are depicted in pink, although other transcription factors may bind the E-boxes. Plus signs indicate an increase of ZRS activity, minus signs represent reduction of activity. The power (on/off) symbol represents that the 3′ conserved region is necessary for ZRS activity. Ets and Etv4/5 binding sites from Lettice et al. (2012); Lim et al. (2024) are shown as green boxes with horizontal lines, and red boxes with diagonal lines, respectively. The sequences associated with Werner mesomelic syndrome (WMS) are shown as a yellow box, the RAR/NFκβ site is shown as a teal box, and the Lhx2 sites are shown as orange boxes with vertical lines.

The conserved 3′ subdomain of the ZRS that was contained within our Fragment 3 (F3) is the only subdomain to retain activity, although it is not sufficient for full ZRS activity. Further, only constructs containing the 3′ subdomain have substantial activity (Figure 1) suggesting it contains binding sites that are necessary for initiation. Indeed, the 3′ subdomain contains three ETS binding sites, a Hox binding site, and an overlapping retinoic acid receptor (RAR)/NFκB/ETS4 site (Figure 6). In addition, we found a critical E-box in the 3′ subdomain (E-box 3) that promotes robust ZRS activity. The importance of this E-box was demonstrated when it was reintroduced into our ZRSΔ5 (ZRSΔ5+E3, Figure 4B) construct and recovered ZRS activity. However, E-box 3 is not essential as its absence does not prevent activity in the full-length ZRS when the functional Hoxd13 binding sites are present (ZRSΔ5+Hx1Hx2, Figure 4A). There is also an ETV binding site within the 3′ subdomain allowing ETV4/5 to recruit histone deacetylases to restrict chromatin access and subsequent activation (Lettice et al., 2012). Nevertheless, the individual and combined 5′ and central fragments (F1, F2, and F1F2) may have little or no activity because they lack E-box 3 and other initiating sites within the 3′ subdomain.

Hand2 has long been recognized as a critical upstream transcription factor for Shh. Our data suggest the 3′ E-box (E-box 3) is key in Shh activation and not the consensus Hand2 binding site found in the central subdomain (E-box 2). This is supported by evidence that deleting the canonical Hand2 binding site does not affect Shh expression, shown by Lettice et al. (2017). Osterwalder and colleagues demonstrated interaction between Hand2 and amplicons containing E-box 2 in e10.5 mice using ChIP-qPCR, though the 5′ E-box (E-box 1) and E-box 3 were not tested (Osterwalder et al., 2014). While these data show Hand2 can bind E-box 2, our data suggest that E-boxes 1 and 2 are repressive, not activating as was previously thought.

Lettice and co-workers also found the 5′ subdomain containing E-box1 lacked activity (their DelD construct) (Lettice et al., 2014). However, in contrast to our data demonstrating F1F3 restored wild type-like activity, they found that when the 5’subdomain was combined with the central subdomain containing E-box 2, (DelB, similar to our F1F2 construct) there was activity similar to wild type, albeit somewhat contracted spatially. Although not quantitated, this may indicate that E-box 2 in the murine model is sufficient to initiate intrinsic activity. The sequence for E-box 3 in the mouse has a base change compared to the chicken and human genome replacing a “C” with an “A” (see Figure 3), which likely alters affinity (Lim et al., 2024). Unfortunately, protein binding microarray data was not available for Hand2, so relative affinity could not be determined. In addition, knock-in experiments with the truncated DelB fragment that lacked the 3′ subdomain were not sufficient to regulate Shh activity and produced a deficient forelimb phenotype similar to Shh loss-of-function knockouts while the hindlimb phenotype was variably reduced. The authors attribute the difference between the transgenic and knock-in findings to a role for the 3′ end in long-range activity. However, an additional interpretation is that the 3′ subdomain is also needed to attain the threshold levels of Shh expression required for limb development.

Basic helix-loop-helix transcription factors, such as Hand2, require dimerization. Koyano-Nakagawa *et al.* showed that Hand2 binds E-box 1 when heterodimerized with E47, but not as a homodimer (Koyano-Nakagawa et al., 2022). Further, Dai and Cserjesi showed that Hand2 can form homodimers, but that only Hand2-E12 heterodimers were transcriptionally active in yeast- and mammalian-two-hybrid systems (Dai and Cserjesi, 2002). Taken together this suggests that Hand2 is capable of binding E-boxes 1 and 2, but at these sites it may not play an activating role (Welscher et al., 2002).

Moreover, Hand2’s role could vary based on its dimerization partner (Firulli et al., 2007). The expression of other E-box binding factors such as Hey1, Snail, and Twist2 overlap the ZPA and thus, may play a role in Shh's regulation. It is also becoming increasingly clear that Hand2 is flexible enough to utilize E-boxes that differ from its consensus motif (Fernandez-Perez et al., 2019), which may depend upon its dimerization partner. Hand2’s ZRS-related dimerization partners remain to be determined.

The 5′ and central subdomains can contribute to ZRS activity but have little to no activity on their own. The 5′ subdomain has a Hoxd13 binding site, an E-box (E-box 1) and two ETS binding sites. These ETS binding sites (ETS0 & ETS1) are critical for overall ZRS activity and are missing in snakes (Kvon et al., 2016; Leal and Cohn, 2016). Interestingly, there is initial Shh expression in pythons that appears to coincide with the induction of Shh by ETV2 (Leal and Cohn, 2016; Koyano-Nakagawa et al., 2022) suggesting that ETS binding sites other than those of the 5′ subdomain can initiate expression but require these ETS sites to amplify or maintain Shh expression. Lettice and co-workers found several ETS binding sites within the 3′ subdomain; ETS3, which is less than 20 bp from E-box3, was important for full activity (Lettice et al., 2012). We found that E-box 1, which is near ETS1, inhibited ZRS activity. However, when a portion of the 5′ subdomain containing the Hoxd13 binding site, ETS0, and ETS1, but lacking E-box 1, was coupled to the 3′ subdomain (F1F3), it was sufficient to recover full ZRS activity (Figure 1) suggesting that a role for the 5′ region is to amplify the activity of the 3′ subdomain.

The central ZRS subdomain includes a recognized five bp inhibitory sequence identified from the human condition Werner mesomelic syndrome (WMS) and is tightly linked to the Hoxd13 site 2. This site has also been implicated by Xu and colleagues as an inhibitory site used by Hox5 paralogs to restrict the anterior activity of the ZRS through interaction with Plzf, whose binding site overlaps the WMS region (Xu et al., 2013). In our Hox binding site affinity analysis, we found that the Hox5 paralogs have a very high relative affinity (0.98-1.00) for a Hox site previously identified as an Lhx2 binding site, though they could also bind to Hoxd13 site 2 (Figure 2). In our studies we found that the central E-box (E-box 2) had an inhibitory effect on activity when present (Figure 4B). In addition to the inhibitory regions, we demonstrate that the Hoxd13 site 2 is activating. Similarly, when Lettice et al. disrupted the Hoxd13 site 2 (labeled as Hoxsite 3) and two adjacent potential Hox sites (Hoxsites1 and 2, see Figure 2) within the central subdomain, activity was reduced supporting a role for Hox-mediated activation (Lettice et al., 2017).

In addition, single nucleotide variations (SNVs) in the central ZRS often lead to anterior ectopic Shh expression; these have been linked to preaxial polydactyly, syndactyly, triphalangeal thumb, and WMS. Remarkably, the majority of clinically significant SNVs result in increased Shh expression either by loss of a repressor or gain of an activator (Bass et al., 2015) (Supplementary Table S1; Supplementary Figure S1). Lim and co-workers demonstrated that subtle increases in ETS binding affinity could extend ZRS activity into the anterior margin (Lim et al., 2024) causing ectopic Shh expression and explaining some SNVs associated with preaxial polydactyly. Repressors such as Etv4 and Etv5 have been reported to inhibit the ZRS anteriorly and localize its expression to the ZPA (Lettice et al., 2017). There is an ETV binding site within the central region and within the 3′ subdomain. These ETVs are thought to recruit histone deacetylases restricting chromatin accessibility and ZRS activity. Taken together, these data suggest the central ZRS subdomain, with both repressive and activating regions, plays a role in fine-tuning the level and localization of Shh expression. The central ZRS subdomain also tends to foster ectopic activity when disrupted.

In our study, we evaluated the isolated ZRS sequence for intrinsic functional domains, however, the genetic context (chromatin folding, associated proteins, and other CRMs) within the regulatory neighborhood (topologically associated domain or TAD), also contributes to the robustness and the regulatory function of the ZRS (Petit et al., 2016; Paliou et al., 2019). For example, loss of two Lhx2 binding sites within the ZRS has no evident effect on intrinsic activity of the isolated ZRS (Bower et al., 2024; Britton et al., 2025), however, in a preliminary report, the loss of these binding sites within their endogenous genetic context disrupts Shh expression producing a phenotype akin to the loss of *Shh* (Bower et al., 2024; Bower and Kvon, 2025). These data suggest that Lhx2 binding confers a benefit for long range enhancer-promoter interactions, tethering it near the Shh promoter. Interestingly, the two canonical Lhx2 binding domains (see Figure 2) and one non-canonical predicted Lhx2 binding site are within the 5′ and central subdomains, suggesting these subdomains are necessary to confer long-range enhancer promoter interactions. This differs from the report by Lettice that suggests that the 3′ subdomain is required for long range interaction (Lettice et al., 2014). This is an area of current study and further work will likely clarify the mechanism of long-range enhancer-promoter tethering within the ZRS.

Our data, combined with previous reports, support a working model of ZRS activity with three modules. First, the 3′ activation subdomain contains an E-box (the likely site of Hand2 binding) and ETS binding sites, of which at least one is likely required for initiation, and a binding site (identified as ETVB) that can toggle ZRS activity on or off depending on whether it is occupied by GABPα or ETV4/5, respectively. Second, the 5′ amplification subdomain contains two ETS binding sites (ETS0 & ETS1) that enhance activity, a Hoxd13 site that enhances activity, and an inhibitory E-box (E-box 1). Finally, the central localization subdomain contains inhibitory sequences, the WMS sequence, E-box 2, Hox5 paralog-Plzf interacting domain, and multiple enhancing Hox sites including a Hoxd13 site. These modules of the ZRS are represented in the diagram in Figure 6 and work collectively to initiate, maintain, and localize Shh expression to the posterior sub-AER mesoderm during limb outgrowth.

## Data Availability

The original contributions presented in the study are included in the article/Supplementary Material, further inquiries can be directed to the corresponding author.
