# Supplementary material for: The 3′ region of the ZPA regulatory sequence (ZRS) is required for activity and contains a critical E-box
Source: Front Cell Dev Biol. 2025 Jul 2;13:1569573. doi: 10.3389/fcell.2025.1569573 (PMC12263586; doi:10.3389/fcell.2025.1569573)
Supplement: Supplementary file 1 [file DataSheet1.pdf]

Supplementary Material

1 Supplementary Data

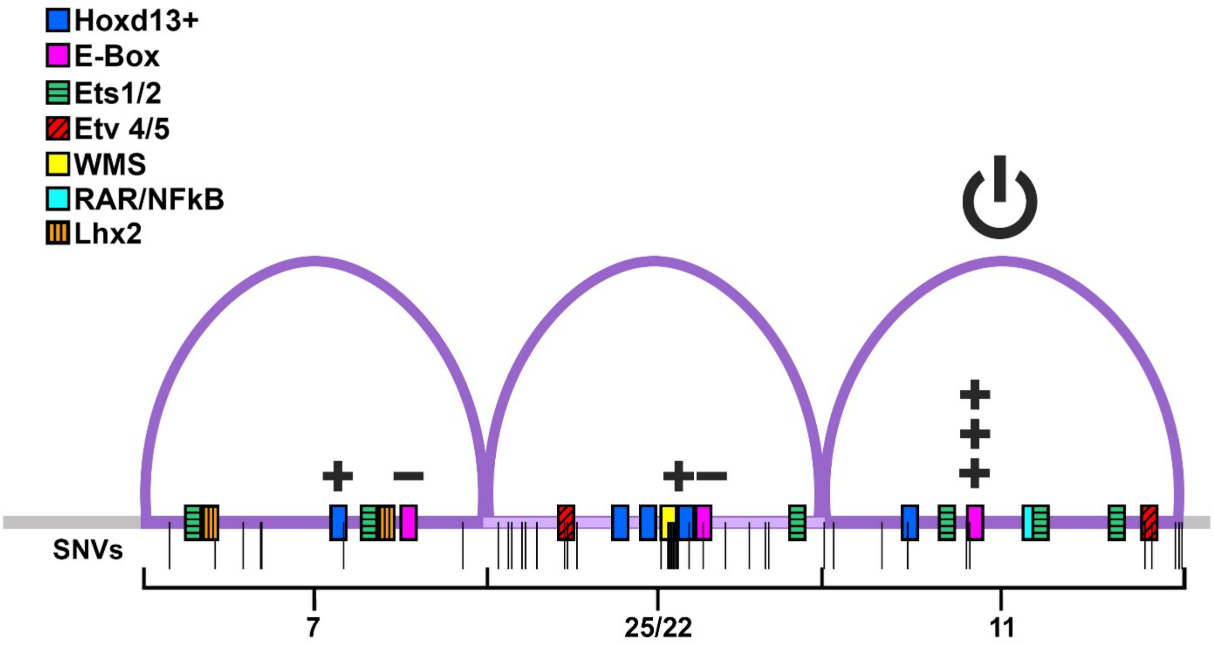

Supplementary Figure 1: Distribution of Reported Single Nucleotide Variations (SNVs) within the ZRS Subdomains. This is a graphic representation of SNVs within the ZRS that are reported to demonstrate preaxial polydactyly or ectopic activity in transgenic mice as listed in Supplementary Table 1. The SNVs are aligned on a schematic of the ZRS subdomains with predicted transcription factor binding sites. The central subdomain contains 25 disruptive SNVs that target 22 different sites, compared with 7 in the 5’ subdomain and 11 in the 3’ subdomain. The distribution suggests an important role for the central subdomain in localizing ZRS activity with the Werner-mesomelic syndrome (WMS) site being a hot spot for pathogenic SNVs (10 SNVs over 7 sites from n401-417) associated with triphalangeal thumbs and preaxial polydactyly. All 9 of the SNVs from this region that have been tested, demonstrated anterior ectopic ZRS activity in transgenic mice.

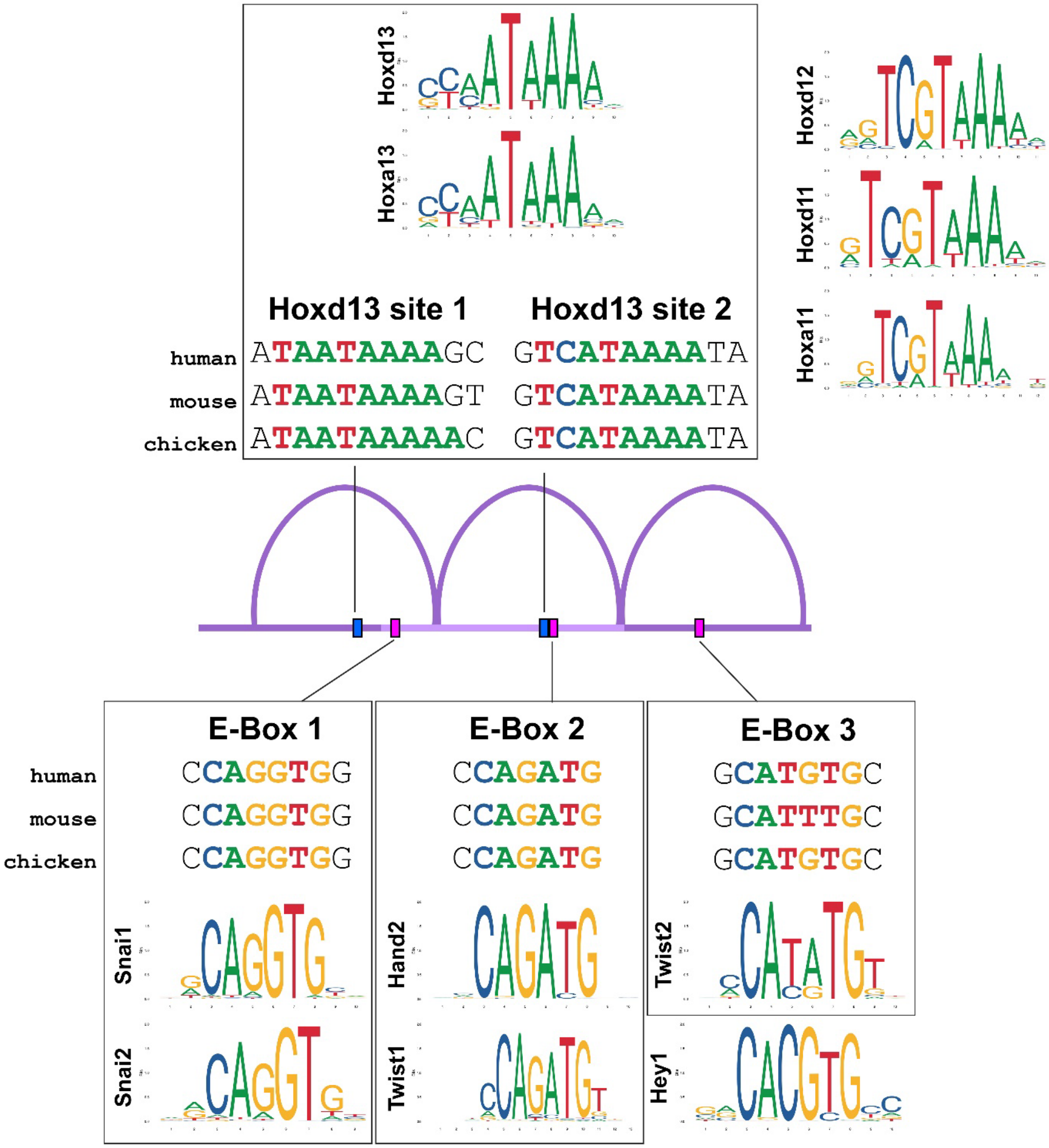

Supplementary Figure 2: Transcription factor consensus binding motifs suggest possible regulators for each ZRS conserved binding site. A ZRS diagram showing the Hox and E-box sequences relevant to this study. Top: Hoxd13's degenerate motif, [C/T][A/C]ATAAA, is found twice in the ZRS at sites we have called Hoxd13 site 1 and 2. It should be noted that Hoxa13's core binding motif is nearly identical to Hoxd13's motif, though Hoxa13 is expressed later in limb development. Other homeodomain factors that could act on the ZRS (Hoxa11, Hoxd11, Hoxd12) are less likely to use these sites due to their consensus motifs. Bottom: Each of the ZRS E-boxes bears a resemblance to the binding motifs of TFs known to be co-expressed with Shh in the limb. In addition to Twist 2's motif, E-box 3 is identical to sequence bound by Hand2/E12 (Dai and Cserjesi, 2002). For ease of comparison, the reverse complements of Hoxd13 site 2 and E-Box 3 are shown. Sequence Logos downloaded from JASPAR (https://jaspar.elixir.no/) (Rauluseviciute et al., 2023).

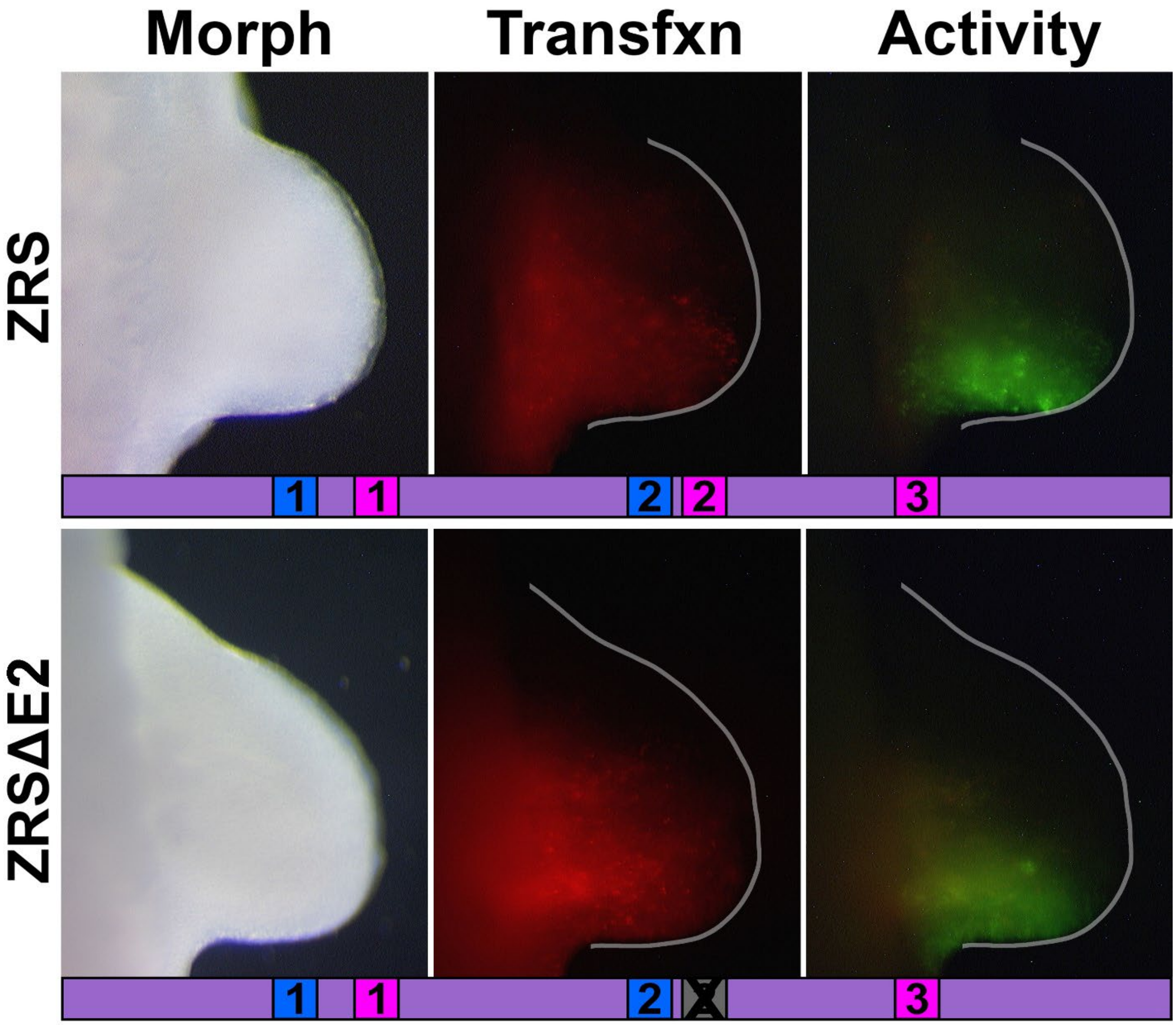

Supplementary Figure 3: ZRS activity persists despite disruption of the central E-box 2 (E2). Hand2 has been shown to bind to the E-box of the central subdomain, E-box 2 (E2), but activity of the ZRS persists even when the E2 binding site is disrupted. This finding is consistent with other reports that indicate that the central domain is not necessary for ZRS activity (Lettice et al., 2017).

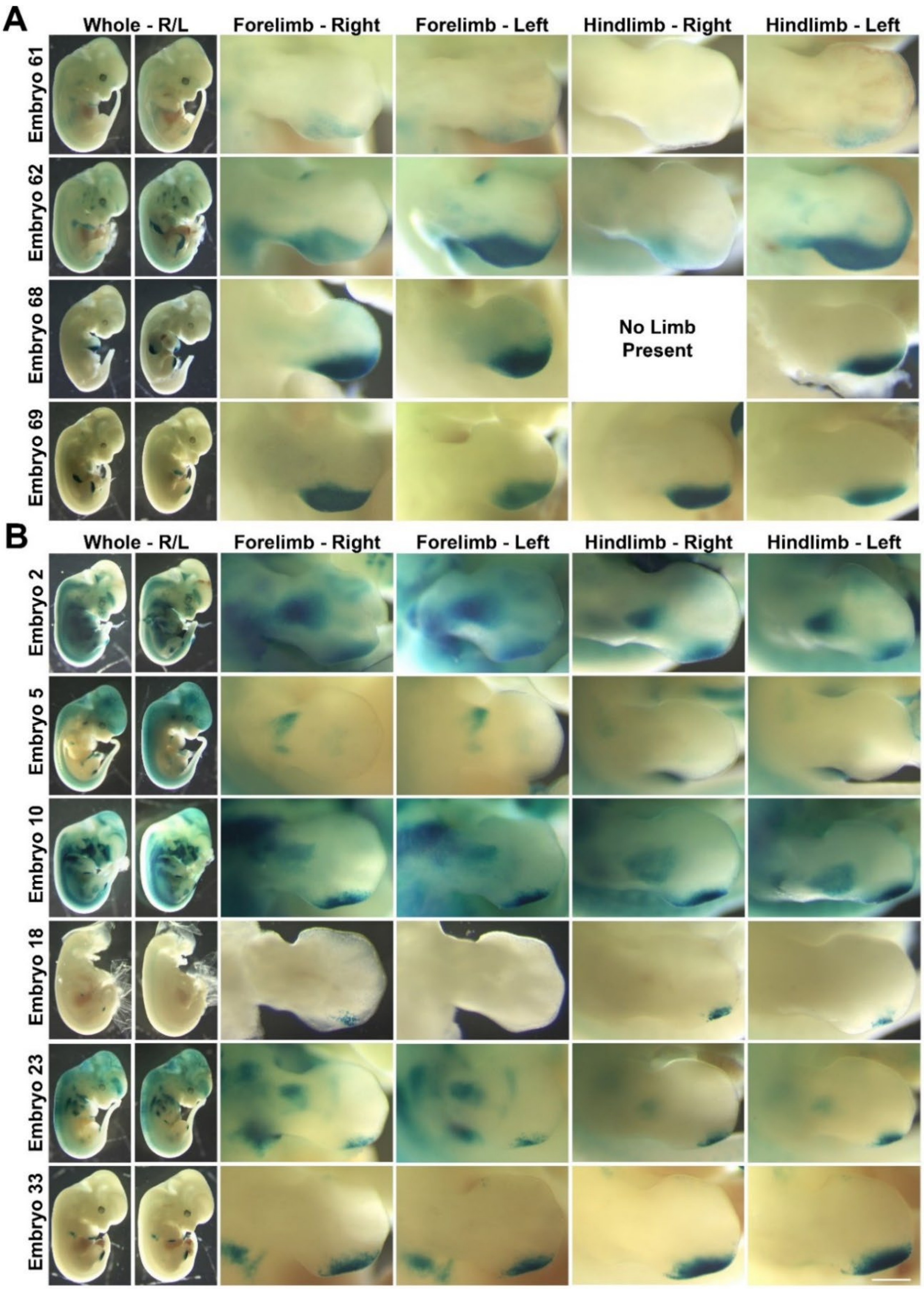

Supplementary Figure 4: Transgenic Mouse Embryos. A) Mouse embryos (e12.5) that were successfully transfected with wild-type hZRS in the HSP68-LacZ plasmid. B) Mouse embryos (e12.5) that were successfully transfected with HSP68-LacZ plasmid harboring hZRSΔ5. Scale bar represents 1mm.

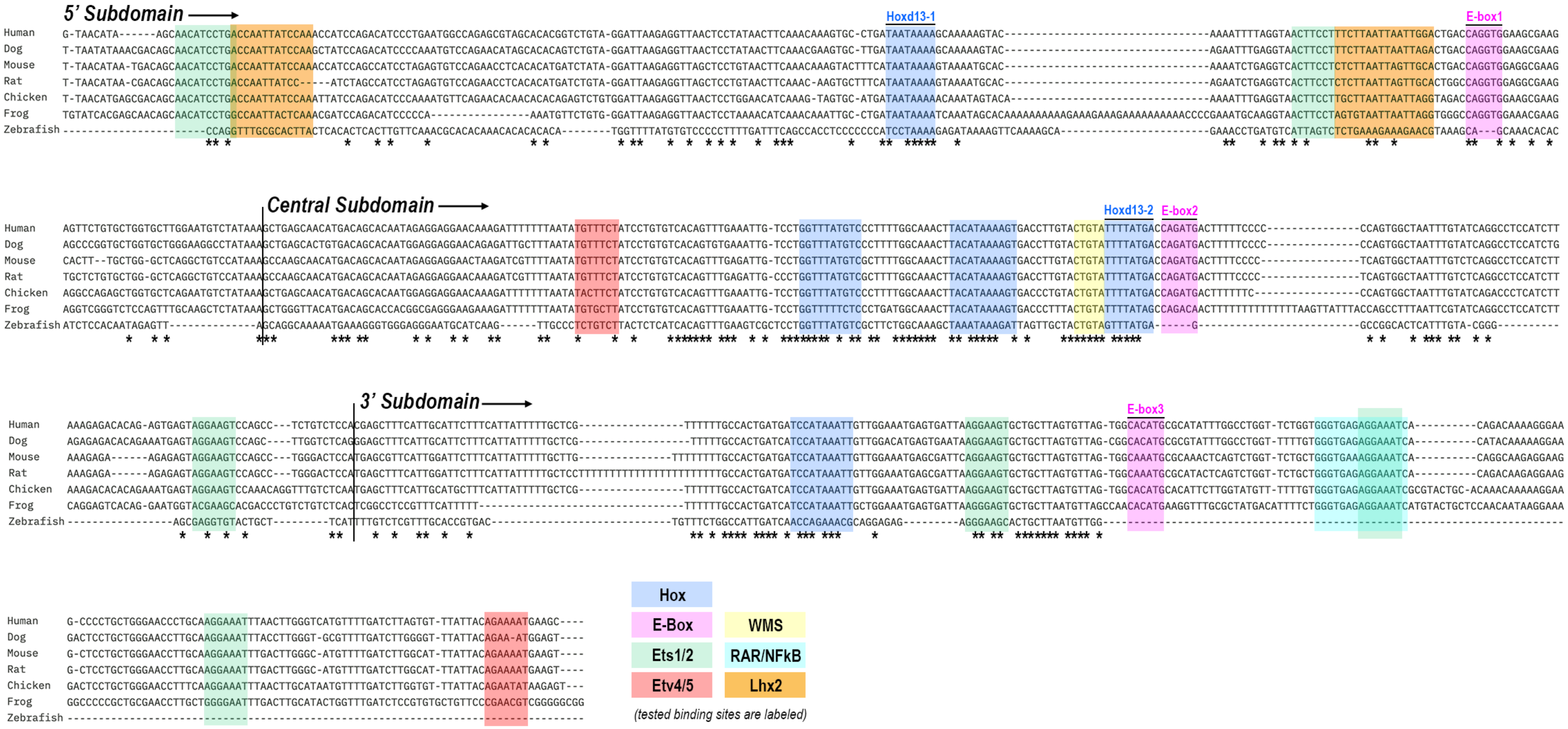

Supplementary Figure 5: ZRS ClustalW Alignment. Multiple sequence alignment of the conserved ZRS region for the following species: human, dog, mouse, rat, chicken, frog, and zebrafish. Alignment was performed using Multiple Alignment using Fast Fourier Transform (MAFFT) (Madeira et al., 2024). Output formatted as ClustalW. Base pairs that are conserved across all species are indicated with an asterisk below the respective base pair. The three ZRS subdomains: 5', central, and 3', are annotated. All binding sites listed in Figure 7 are color annotated, with the name of tested binding sites annotated above the respective site. Hox: blue, E-box: pink, Etv4/5: red, WMS: yellow, Ets1/2 : green, RAR/NFkB: turquoise.

2 Supplementary Tables

2.1 Supplementary Table 1 (xlsx file)

ZRS Single Nucleotide Variations (SNVs). A table of all ZRS SNVs documented to date, including subdomain location, references, and phenotypes. CD: clinodactyly, ED: ectrodactyly, HF: hypoplastic fibula, HH: hypoplastic hallux, HP: hypoplastic pollex, HR: hypoplastic radius, HT: hypoplastic tibia, HU: hypoplastic ulna, PAP: postaxial polydactyly, PPD: preaxial polydactyly, SD: syndactyly, TH: thenar hypoplasia, TPT: triphalangeal thumb, WMS: Werner-mesomelic syndrome.

**CHICKEN ZRS FRAGMENT SEQUENCES**		
**Color Legend**		
**Subdomains**	**Hoxd13 Binding Sites**	**E-Box Binding Sites**
5'Subdomain<br>Central Subdomain<br>3' Subdomain<br>additional chicken-specific region	**Hoxd13-1**<br>**Hoxd13-2**	***Ebox1***<br>***Ebox2***<br>***Ebox3***

Chicken ZRS F1 – 749bp: 581 bp of adjacent upstream DNA + 168 bp of 5′ subdomain

```
>Chicken ZRS F1
atatgttagaattgaaaactttttgcatgccttcataaatttttgtctctgtggagtctttacatgaagttgtattttgtgtttagatcctctgacagttcttt
gtcatgggccttcatgttgtgtattacaactatgaaacaaaaaaggtgtcagctgtgttcattcatgtcaaaattaacctttagtgctctaaaagaggaaactg
ttatttgtagttgagcatttatagtttgcagtgctgatgtttccatgtgtgataaggctgcacagagttacacgaatgctgctgtgtgaggaaggcaagcattt
ttgaagagataattgctgtaccttccttgaccttaaaatgtgctttctcttgatgtaacttcaactgaaatttgtatttgtttgaagatgacaaagataaagat
gtttaggagtaatttccttgtaagaaaatacagaagtgcgatttaccatcgtgagaaactggtataactgggtattgcataatcaggagtgtagagttttaaaa
aggctttatgaagtatttaccttaatactgatctttgaactcgaagtcacggcataaagtTTAACATGAGCGACAGCAACATCCTGACCAATTATCCAAATTA
TCCAGACATCCCAAAATGTTCAGAACACAACACACAGAGTCTGTGGATTAAGAGGTTAACTCCTGGAACATCAAAGTAGTGCATGATAATAAAAACAAATAGTA
CAAAAATTTGAGGTAACTTCC
```

Chicken ZRS F2 – 309bp: 60 bp of 5’ subdomain + full central subdomain (236bp) + 13 bp of 3’ subdomain

>Chicken ZRS F2
TAATTAGGTAGAC***CAGGTG***GAAGCGAAGAGGCCAGAGCTGGTGCTCAGAATGTCTATAAAGCTGAGCAACATGACAGCACAATGGAGGAGGAACAAAGA
TTTTTTTAATATACTTCTATCCTGTGTCACAGTTTGAAATTGTCCTGGTTTATGTCCCTTTTGGCAAACTTACATAAAAGTGACCCTGTACTGTA**TTTTATGA**
C***CAGATG***ACTTTTTTCCCAGTGGCTAATTTGTATCAGACCCTCATCTTAAAGACACACAGAAATGAGTAGGAAGTCCAAACAGGTTTGTCTCAATGAGCTTT
CAT

Chicken ZRS F3 – 302bp: 236 bp of 3’ subdomain + 66bp of adjacent downstream DNA

>Chicken ZRS F3
TCATTATTTTTGCTCGTTTTTTGCCACTGATCATCCATAAATTGTTGGAAATGAGTGATTAAGGAAGTGCTGCTTAGTGTTAGTGG***CACATG***CACATTCTTGG
TATGTTTTTTGTGGGTGAGAGGAAATCGCGTACTGCACAAACAAAAAGGAAGACTCCTGCTGGGAACCTTTCAAGGAAATTTAACTTGCATAATGTTTTGA
TCTTGGTGTTTATTACAGAATATAAGAGTaatatttcaccagctattgttatgtgtcagctaggctctccgtctgctttgtcattccttgatgtc

Chicken ZRS F1F2 – 1065bp: 581 bp of adjacent upstream DNA + full 5’ subdomain + full central subdomain + 11bp of 3’ subdomain

>Chicken ZRS F1F2
atatgttagaattgaaaacttttgcatgccttcataaatttttgtctctgtggagtctttacatgaagttgtattttgtgtttagatcctctgacagttctttgtcatgggcct
tcatgttgtgtattacaactatgaaacaaaaaaggtgtcagctgtgttcattcatgtcaaaattaacctttagtgctctaaaagaggaaactgttatttgtagttgagcatttat
agtttgcagtgctgatgtttccatgtgtgataaggctgcacagagttacacgaatgctgctgtgtgaggaaggcaagcatttttgaagagataattgctgtaccttccttgacct
taaaatgtgctttctcttgatgtaacttcaactgaaatttgtatttgtttgaagatgacaaagataaagatgtttaggagtaatttccttgtaagaaaatacagaagtgcgattt
accatcgtgagaaactggtataactgggtattgcataatcaggagtgtagagttttaaaaaggctttatgaagtattttaccttaatactgatctttgaactcgaagtcacggca
taaagtTTAACATGAGCGACAGCAACATCCTGACCAATTATCCAAATTATCCAGACATCCCAAAATGTTCAGAACACAACACACAGAGTCTGTGGATTAAG
AGGTTAACTCCTGGAACATCAAAGTAGTGCATGA**TAATAAAA**ACAAATAGTACAAAAATTTGAGGTAACTTCCTTGCTTAATTAATTAGGTAGAC***CAGGTG***
GAAGCGAAGAGGCCAGAGCTGGTGCTCAGAATGTCTATAAAGCTGAGCAACATGACAGCACAATGGAGGAGGAACAAAGATTTTTTTAATATACTTCTAT
CCTGTGTCACAGTTTGAAATTGTCCTGGTTTATGTCCCTTTTGGCAAACTTACATAAAAGTGACCCTGTACTGTA**TTTTATGA**C***CAGATG***ACTTTTTTCCCAG
TGGCTAATTTGTATCAGACCCTCATCTTAAAGACACACAGAAATGAGTAGGAAGTCCAAACAGGTTTGTCTCAATGAGCTTTCAT

Chicken ZRS F2F3 – 615bp: 60 bp 5’ subdomain + full central subdomain + full 3’ subdomain + 66bp of adjacent downstream DNA

>Chicken ZRS F2F3
TAATTAGGTAGAC***CAGGTG***GAAGCGAAGAGGCCAGAGCTGGTGCTCAGAATGTCTATAAAGCTGAGCAACATGACAGCACAATGGAGGAGGAACAAAGA
TTTTTTTAATATACTTCTATCCTGTGTCACAGTTTGAAATTGTCCTGGTTTATGTCCCTTTTGGCAAACTTACATAAAAGTGACCCTGTACTGTA**TTTTATGA**
C***CAGATG***ACTTTTTTCCCAGTGGCTAATTTGTATCAGACCCTCATCTTAAAGACACACAGAAATGAGTAGGAAGTCCAAACAGGTTTGTCTCAATGAGCTTT
CATTGCATGCTTTCATTATTTTTGCTCGTTTTTTGCCACTGATCATCCATAAATTGTTGGAAATGAGTGATTAAGGAAGTGCTGCTTAGTGTTAGTGG***CACAT***
GCACATTCTTGGTATGTTTTTTGTGGGTGAGAGGAAATCGCGTACTGCACAAACAAAAAGGAAGACTCCTGCTGGGAACCTTTCAAGGAAATTTAACTTGC
ATAATGTTTTGATCTTGGTGTTTATTACAGAATATAAGAGTaatatttcaccagctattgttatgtgtcagctaggctctccgtctgctttgtcattccttgatgtc

Chicken ZRS F1F3 – 1058bp: 581 bp of adjacent upstream DNA + full 5’ subdomain + full central subdomain + full 3’ subdomain + 66bp of adjacent downstream DNA

>Chicken ZRS F1F3
atatgttagaattgaaaacttttgcatgccttcataaatttttttgtctctgtggagtctttacatgaagttgtattttgtgtttagatcctctgacagttctttgtcatgggcct
tcatgttgtgtattacaactatgaaacaaaaaaggtgtcagctgtgttcattcatgtcaaaattaacctttagtgctctaaaagaggaaactgttatttgtagttgagcatttat
agtttgcagtgctgatgtttccatgtgtgataaggctgcacagagttacacgaatgctgctgtgtgaggaaggcaagcattttttgaagagataattgctgtaccttccttgacct
taaaatgtgctttctcttgatgtaacttcaactgaaatttgtatttgtttgaagatgacaaagataaagatgtttaggagtaatttccttgtaagaaaatacagaagtgcgattt
accatcgtgagaaactggtataactgggtattgcataatcaggagtgtagagttttaaaaaggctttatgaagtattttaccttaatactgatctttgaactcgaagtcacggca
taaagtTTAACATGAGCGACAGCAACATCCTGACCAATTATCCAAATTATCCAGACATCCCAAAATGTTCAGAACACAACACACAGAGTCTGTGGATTAAG
AGGTTAACTCCTGGAACATCAAAGTAGTGCATGA**TAATAAAA**ACAAATAGTACAAAAATTTGAGGTAACTTCCTTGCTTAATTAATTAGGTAGAC***CAGGTG***
GAAGCGAAGAGGCCAGAGCTGGTGCTCAGAATGTCTATAAAGCTGAGCAACATGACAGCACAATGGAGGAGGAACAAAGATTTTTTTAATATACTTCTAT
CCTGTGTCACAGTTTGAAATTGTCCTGGTTTATGTCCCTTTTGGCAAACTTACATAAAAGTGACCCTGTACTGTA**TTTTATGA**C***CAGATG***ACTTTTTTCCCAG
TGGCTAATTTGTATCAGACCCTCATCTTAAAGACACACAGAAATGAGTAGGAAGTCCAAACAGGTTTGTCTCAATGAGCTTTCATTGCATGCTTTCATTATT
TTTGCTCGTTTTTTGCCACTGATCATCCATAAATTGTTGGAAATGAGTGATTAAGGAAGTGCTGCTTAGTGTTAGTGG***CACATG***CACATTCTTGGTATGTTTT
TTGTGGGTGAGAGGAAATCGCGTACTGCACAAACAAAAAGGAAGACTCCTGCTGGGAACCTTTCAAGGAAATTTAACTTGCATAATGTTTTGATCTTGGTG
TTTATTACAGAATATAAGAGTaatatttcaccagctattgttatgtgtcagctaggctctccgtctgctttgtcattccttgatgtc

2.2 Supplementary Table 2

Chicken ZRS Sequence Annotation. A list of the sequences for fragments F1, F2, F3, F1F2, F2F3, and F1F3. Sequences are presented in FASTA format, and annotated by color to demarcate subdomains, wherein the 5’ subdomain is purple, the central subdomain is orange, and the 3’ subdomain is blue. Hoxd13 binding sites have been indicated in bold and underline, while E-box binding sites are bold, underlined, and italicized. A breakdown of base pairs and the subdomains they overlap are also provided for each fragment. Sequence was extracted based on Galgal6. A table of the assemblies and coordinates (along with coordinates for each subdomain) used can be found in Supplementary Table 5.

2.3 Supplementary Table 3 (xlsx file)

Primers used in this study. All primers are listed and were made by Integrated DNA Technologies Inc., (Coralville, Iowa). Note that the cZRS construct was generated by first pulling-down a larger fragment that included the pre-ZRS with the listed primers, then a truncated version of was generated using the Erase-a-Base system.

Chicken Embryo Limb Inclusion Criteria

Score	0	1	2	3	4	5
**Morphology**	Limb is absent or indistinguishable from surrounding tissue	Limb is present but severely malformed or damaged from TREP or harvest, anatomical axes may be unclear.	Limb may be moderately malformed, anatomical axes are clear, limb is less than 50% the size of the contralateral limb.	Limb may be minimally malformed, anatomical axes are clear, limb is at least 50% the size of the contralateral limb.	Limb is fully formed, anatomical axes are clear, size is at least 75% of the contralateral limb, may have superficial defects.	Limb is fully formed, anatomical axes are clear, size is approximately equal to the contralateral limb, no obvious defects.
**Transfection efficiency**	No RFP is visible above background.	Only faint RFP is visible.	RFP is visible but may be faint or may not cover the target transfection region.	RFP is clearly visible and covers most of the target region.	RFP is bright and covers the entire target region	RFP is very bright and covers the entire target region
**Auto-fluorescence**	An extreme amount of autofluorescence is visible	An excessive amount of autofluorescence is visible	A moderate amount of autofluorescence is visible	A minimal amount of autofluorescence is visible	A very minimal amount of autofluorescence is visible	No autofluorescence is visible

2.4 Supplementary Table 4

Chicken Embryo Limb Inclusion Criteria Description: Embryo limbs were ranked on a 0-5 scale for morphology, transfection based on RFP, and autofluorescence. Limbs needed to score a 3 or above in each category to be included in our analysis.

Morphology: TREP can cause significant damage to limb tissue making it difficult to assess transfection quality or enhancer activity pattern.

Transfection: Transfection coverage and quality can vary due to many factors involved in TREP such as subtle differences in injection location, DNA solution leaking out of the coelom before the current is applied, and differences in electrical conductivity during electroporation.

Autofluorescence:Light in a fluorescent image that 1) can be outside the transfected region, 2) can appear yellow-green in a GFP image and can result from an embryo that was dead prior to harvest, or an embryo that was not imaged quickly enough after harvest.

ZRS SUBDOMAIN COORDINATES					
	**Assembly**	**Total bp**	**5' Subdomain Coordinates**	**Central Subdomain Coordinates**	**3' Subdomain Coordinates**
Human	Hg38	706bp	chr7: 156,791,604-156,791,833	chr7: 156,791,370-156,791,603	chr7: 156,791,128-156,791,369
Dog	canFam3	710bp	chr16: 19,379,383-19,379,618	chr16: 19,379,149-19,379,382	chr16: 19,378,909-19,379,148
Mouse	mm10	704bp	chr5: 29,315,393-29,315,625	chr5: 29,315,165-29,315,392	chr5: 29,314,922-29,315,164
Rat	rn7	715bp	chr4: 6,063,705-6,063,933	chr4: 6,063,934-6,064,161	chr4: 6,064,162-6,064,419
Chicken	Galgal6	726bp	chr2: 8,553,715-8,553,951	chr2: 8,553,479-8,553,714	chr2: 8,553,226-8,553,478
Frog	xenTro10	750bp	chr6: 17,346,614-17,346,867	chr6: 17,346,362-17,346,613	chr6: 17,346,118-17,346,361
Zebrafish	danRer10	461bp	chr7: 40,398,711-40,398,906	chr7: 40,398,907-40,399,084	chr7: 40,399,085-40,399,171

Red = ZRS on reverse (-) strand

2.5 Supplementary Table 5

ZRS Subdomain Coordinates: A list of the ZRS coordinates for all species evaluated in conservation analysis. ZRS coordinates for the 5', Central, and 3' subdomains are listed, in addition to the utilized assembly number and total base pair size. Evaluated species include human, dog, mouse, rat, chicken, frog, and zebrafish. Species listed in red are those with the ZRS on the reverse (-) strand.

**BINDING SITE LOCATIONS + SEQUENCES**<br>***Variable binding site**						
**Species**	**Assembly**	**5' Subdomain**		**Central Subdomain**		**3' Subdomain**
		**Hoxd13-1**	**E-box1**	**Hoxd13-2**	**E-box2**	**E-box3**
Human	Hg38	chr7: 156,791,703-156,791,710 TAATAAAA	chr7: 156,791,645-156,791,650 CAGGTG	chr7: 156,791,462-156,791,469 TTTTATGA	chr7: 156,791,455-156,791,460 CAGATG	chr7: 156,791,258-156,791,263 CACATG
Dog	canFam3	chr16: 19,379,482-19,379,489 TAATAAAA	chr16: 19,379,424-19,379,429 CAGGTG	chr16: 19,379,241-19,379,248 TTTTATGA	chr16: 19,379,234-19,379,239 CAGATG	chr16: 19,379,038-19,379,043 CACATG
Mouse	mm10	chr5: 29,315,489-29,315,496 TAATAAAA	chr5: 29,315,431-29,315,436 CAGGTG	chr5: 29,315,251-29,315,258 TTTTATGA	chr5: 29,315,244-29,315,249 CAGATG	**chr5: 29,315,051-29,315,056 CAAATG**
Rat	rn7	chr4:6,063,828-6,063,835 TAATAAAA	chr4:6,063,888-6,063,893 CAGGTG	chr4:6,064,068-6,064,075 TTTTATGA	chr4:6,064,077-6,064,082 CAGATG	**chr4:6,064,285-6,064,290 CAAATG**
Chicken	Galgal6	chr2: 8,553,815-8,553,822 TAATAAAA	chr2: 8,553,756-8,553,761 CAGGTG	chr2: 8,553,573-8,553,580 TTTTATGA	chr2: 8,553,566-8,553,571 CAGATG	chr2: 8,553,367-8,553,372 CACATG
Frog	xenTro10	chr6: 17,346,746-17,346,753 TAATAAAA	chr6: 17,346,655-17,346,660 CAGGTG	**chr6: 17,346,472-17,346,479 TTTTATAG**	**chr6: 17,346,465-17,346,470 CAGACA**	chr6: 17,346,265-17,346,270 CACATG
Zebrafish	danRer10	**chr7: 40,398,812-40,398,819 TCCTAAAA**	**chr7: 40,398,866-40,398,871 CA---G**	**chr7: 40,399,040-40,399,047 GTTTATGA**	**chr7: 40,399,048-40,399,052 -----G**	**chr7:40,399,172-40,399,175 N/A**

2.6 Supplementary Table 6

Hoxd13 and E-box Binding Site Coordinates and Sequences. List of conserved Hoxd13 and E-box binding sites within the 5', central, and 3' subdomains across species. Sequences, along with respective coordinates and assemblies used for each species, are listed. Evaluated species: human, dog, mouse, rat, chicken, frog, and zebrafish. Binding sites with variable sequences are indicated in red. Note that Mouse and Rat share

FULL ZRS SEQUENCES

Color/Binding site Key:

Purpose = 5’ subdomain Orange = Central subdomain Blue = 3’ subdomain

Hoxd13 ***E-box***

>Human

GTAACATAAGCAACATCCTGACCAATTATCCAAACCATCCAGACATCCCTGAATGGCCAGAGCGTAGCACACGGTCTGTAGG
ATTAAGAGGTTAACTCCTATAACTTCAAACAAAGTGCCTGA**TAATAAAA**GCAAAAAGTACAAAATTTTAGGTAACTTCCTTT
CTTAATTAATTGGACTGAC***CAGGTG***GAAGCGAAGAGTTCTGTGCTGGTGCTTGGAATGTCTATAAAGCTGAGCAACATGACA
GCACAATAGAGGAGGAACAAAGATTTTTTTAATATGTTTCTATCCTGTGTCACAGTTTGAAATTGTCCTGGTTTATGTCCCTTT
TGGCAAACTTACATAAAAGTGACCTTGTACTGTA**TTTTATGA**C***CAGATG***ACTTTTTCCCCCCAGTGGCTAATTTGTATCAGGC
CTCCATCTTAAAGAGACACAGAGTGAGTAGGAAGTCCAGCCTCTGTCTCCACGAGCTTTCATTGCATTCTTTCATTATTTTTGC
TCGTTTTTTGCCACTGATGATCCATAAATTGTTGGAAATGAGTGATTAAGGAAGTGCTGCTTAGTGTTAGTGG***CACATG***CGCA
TATTTGGCCTGGTTCTGGTGGGTGAGAGGAAATCACAGACAAAAGGGAAGCCCCTGCTGGGAACCCTGCAAGGAAATTTAAC
TTGGGTCATGTTTTGATCTTAGTGTTTATTACAGAAAATGAAGC

>Dog

TTAATATAAACGACAGCAACATCCTGACCAATTATCCAAGCTATCCAGACATCCCCAAATGTCCAGAACATAGCACACAGTC
TGTAGGATTAAGAGGTTAACTCCTATAACTTCAAACGAAGTGCTTGA**TAATAAAA**GCAAAAAGTACAGAATTTGAGGTAACT
TCCTTTCTTAATTAATTAGACTGAC***CAGGTG***GAAGCGAAGAGCCCGGTGCTGGTGCTGGGAAGGCCTATAAAGCTGAGCACT
GTGACAGCACAATGGAGGAGGAACAGAGATTGCTTTAATATGTTTCTATCCTGTGTCACAGTGTGAAATTGTCCTGGTTTATG
TCCCTTTTGGCAAACTTACATAAAAGTGACCTTGTACTGTA**TTTTATGA**C***CAGATG***ACTTTTTCCCCCCAGTGGCTAATTTGTA
TCAGGCCTCCATCTGAGAGAGACACAGAAATGAGTAGGAAGTCCAGCTTGGTCTCAGGGAGCTTTCATTGCATTCTTTCATTA
TTTTTGCTCGTTTTTGCCACTGATCATCCATAAATTGTTGGACATGAGTGAATAAGGAAGTGCTGCTTAGTGTTAGCGG***CACAT***
GCGCGTATTTGGCCTGGTTTTTGTGGGTGAGAGGAAATCACATACAAAAAGGAAGACTCCTGCTGGGAACCTTGCAAGGAAA
TTTACCTTGGGTGCGTTTTGATCTTGGGGTTTATTACAGAAATGGAG

>Mouse
TTAACATAATGACAGCAACATCCTGACCAATTATCCAAACCATCCAGCCATCCTAGAGTGTCCAGAACCTCACACATGATCTA
TAGGATTAAGAGGTTAGCTCCTGTAACTTCAAACAAAGTACTTTCA**TAATAAAA**GTAAAATGCACAAAATCTGAGGTCACTT
CCTCTCTTAATTAGTTGCACTGAC***CAGGTG***GAGGCGAAGCACTTTGCTGGGCTCAGGCTGTCCATAAAGCCAAGCAACATGA
CAGCACAATAGAGGAGGAACTAAGATCGTTTTAATATGTTTCTATCCTGTGTCACAGTTTGAGATTGTCCTGGTTTATGTCGC
TTTTGGCAAACTTACATAAAAGTGACCTTGTACTGTA**TTTTATGA**C***CAGATG***ACTTTTCCCCTCAGTGGCTAATTTGTCTCAGG
CCTCCATCTTAAAGAGAAGAGAGTAGGAAGTCCAGCCTGGGACTCCATGAGCGTTCATTGGATTCTTTCATTATTTTTGCTTG
TTTTTTTTGCCACTGATGATCCATAAATTGTTGGAAATGAGCGATTCAGGAAGTGCTGCTTAGTGTTAGTGG***CAAATG***CGCAA
ACTCAGTCTGGTTCTGCTGGGTGAAAGGAAATCACAGGCAAGAGGAAGGCTCCTGCTGGGAACCTTGCAAGGAAATTTGACT
TGGGCATGTTTTGATCTTGGCATTTATTACAGAAAATGAAGT

>Rat
TTAACATAACGACAGCAACATCCTGACCAATTATCCATCTAGCCATCCTAGAGTGTCCAGAACCTCACACATGATCTGTAGGA
TTAAGAGGTTAGCTCCTGTAACTTCAAACAAGTGCTTTCA**TAATAAAA**GTAAAATGCACAGAATCTGAGGTCACTTCCTCTCT
TAATTAGTTGCACTGGC***CAGGTG***GAGGCGAAGTGCTCTGTGCTGGGCTCAGGCTGTCCATAAAGCCAAGCAACATGACAGCA
CAATAGAGGAGGAACAAAGATCGTTTTAATATGTTTCTATCCTGTGTCACAGTTTGAGATTGCCCTGGTTTATGTCGCTTTTGG
CAAACTTACATAAAAGTGACCTTGTACTGTA**TTTTATGA**C***CAGATG***ACTTTTCCCCTCAGTGGCTAATTTGTCTCAGGCCTCC
ATCTTAAAGAGAAGAGAGTAGGAAGTCCAGCCTGGGACTCCATGAGCTTTCATTGGATTCTTTCATTATTTTTGCTCCTTTTTT
TTTTTTTTTTTTTTTTTGCCACTGATGATCCATAAATTGTTGGAAATGAGCGATTAAGGAAGTGCTGCTTAGTGTTAGTGG***CAA***
ATGCGCATACTCAGTCTGGTTCTGCTGGGTGAGAGGAAATCACAGACAAGAGGAAGGCTCCTGCTGGGAACCTTGCAAGGAA
ATTTGACTTGGGCATGTTTTGATCTTGGCATTTATTACAGAAAATGAAGT

>Chicken
TTAACATGAGCGACAGCAACATCCTGACCAATTATCCAAATTATCCAGACATCCCAAAATGTTCAGAACACAACACACAGAG
TCTGTGGATTAAGAGGTTAACTCCTGGAACATCAAAGTAGTGCATGA**TAATAAAA**ACAAATAGTACAAAAATTTGAGGTAAC
TTCCTTGCTTAATTAATTAGGTAGAC***CAGGTG***GAAGCGAAGAGGCCAGAGCTGGTGCTCAGAATGTCTATAAAGCTGAGCAA
CATGACAGCACAATGGAGGAGGAACAAAGATTTTTTTAATATACTTCTATCCTGTGTCACAGTTTGAAATTGTCCTGGTTTAT
GTCCCTTTTGGCAAACTTACATAAAAGTGACCCTGTACTGTA**TTTTATGA**C***CAGATG***ACTTTTTTCCCAGTGGCTAATTTGTAT
CAGACCCTCATCTTAAAGACACACAGAAATGAGTAGGAAGTCCAAACAGGTTTGTCTCAATGAGCTTTCATTGCATGCTTTCA
TTATTTTTGCTCGTTTTTTGCCACTGATCATCCATAAATTGTTGGAAATGAGTGATTAAGGAAGTGCTGCTTAGTGTTAGTGG***C***

ACATGCACATTCTTGGTATGTTTTTTGTGGGTGAGAGGAAATCGCGTACTGCACAAACAAAAAGGAAGACTCCTGCTGGGAA
CCTTTCAAGGAAATTTAACTTGCATAATGTTTTGATCTTGGTGTTTATTACAGAATATAAGAGT

>Frog
TGTATCACGAGCAACAGCAACATCCTGGCCAATTACTCAAACGATCCAGACATCCCCCAAAATGTTCTGTGGGATTAAGAGG
TTAACTCCTAAAACATCAAACAAATTGCCTGA**TAATAAAA**TCAAATAGCACAAAAAAAAAAAGAAAGAAAGAAAAAAAAAA
CCCCGAAATGCAAGGTAACTTCCTAGTGTAATTAATTAGGTGGGC***CAGGTG***GAAACGAAGAGGTCGGGTCTCCAGTTTGCAA
GCTCTATAAAGCTGGGTTACATGACAGCACCACGGCGAGGGAAGAAAGATTTTTTTAATATGTGCTTATCCTGTGTCACAGTT
TGAAATTGTCCTGGTTTTCTCCCTGATGGCAAACTTACATAAAAGTGACCCTTTACTGTA**TTTTATAG**C***CAGACA***ACTTTTTT
TTTTTTTTAAGTTATTTACCAGCCTTTAATTCGTATCAGGCCTCCATCTTCAGGAGTCACAGGAATGGTACGAAGCACGACCCT
GTCTGTCTCACTCGGCCTCCGTTTCATTTTTTTTTTTGCCACTGATCATCCATAAATTGCTGGAAATGAGTGATTAAGGGAGTG
CTGCTTAATGTTAGCCAA***CACATG***AAGGTTTGCGCTATGACATTTTCTGGGTGAGAGGAAATCATGTACTGCTCCAACAATAA
GGAAAGGCCCCCGCTGCGAACCTTGCTGGGGAATTTGACTTGCATACTGGTTTGATCTCCGTGTGCTGTTCCCGAACGTCGGG
GGCGG

>Zebrafish
CCAGGTTTGCGCACTTACTCACACTCACTTGTTCAAACGCACACAAACACACACACATGGTTTTATGTGTCCCCCTTTTGATTT
CAGCCACCTCCCCCCCA**TCCTAAAA**GAGATAAAAGTTCAAAAGCAGAAACCTGATGTCATTAGTCTCTGAAAGAAAGAACG
TAAAG***CAGC***AAACACACATCTCCACAATAGAGTTAGCAGGCAAAAATGAAAGGGTGGGAGGGAATGCATCAAGTTGCCCTC
TGTCTTACTCTCATCACAGTTTGAAGTCGCTCCTGGTTTATGTCGCTTCTGGCAAAGCTAAATAAAGATTAGTTGCTACTGTA**G**
TTTATGA***G***GCCGGCACTCATTTGTACGGGAGCGAGGTGTACTGCTTCATTTTGTCTCGTTTGCACCGTGACTGTTTCTGGCCA
TTGATCAACCAGAAACGCAGGAGAGAGGGAAGCACTGCTTAATGTTGG

2.7 Supplementary Table 7

ZRS Sequences. A list of the full ZRS sequence for the following species: human, dog, mouse, rat, chicken, frog, and zebrafish. A table of the assemblies and coordinates (along with coordinates for each subdomain and binding site) used for each species can be found in Supplementary Tables 6 & 7. Sequences have been annotated by color to demarcate subdomain, wherein the 5' subdomain is purple, the Central subdomain is orange, and the 3' subdomain is blue. Hoxd13 binding sites have been indicated in bold and in underline, while E-box binding sites are bold, underlined, and italicized. Note that in Zebrafish, there is no gap between the Hoxd13-2 and E-box2 binding sites, as the E-box2 in Zebrafish is 1bp. E-box3 is also not present in Zebrafish.

Works Cited

Dai, Y.-S., and Cserjesi, P. (2002). The Basic Helix-Loop-Helix Factor, HAND2, Functions as a Transcriptional Activator by Binding to E-boxes as a Heterodimer. *J Biol Chem* 277, 12604–12612. doi: 10.1074/jbc.m200283200

Lettice, L. A., Devenney, P., Angelis, C. D., and Hill, R. E. (2017). The Conserved Sonic Hedgehog Limb Enhancer Consists of Discrete Functional Elements that Regulate Precise Spatial Expression. *Cell Reports* 20, 1396–1408. doi: 10.1016/j.celrep.2017.07.037

Madeira, F., Madhusoodanan, N., Lee, J., Eusebi, A., Niewielska, A., Tivey, A. R. N., et al. (2024). The EMBL-EBI Job Dispatcher sequence analysis tools framework in 2024. *Nucleic Acids Res.* 52, W521–W525. doi: 10.1093/nar/gkae241

Rauluseviciute, I., Riudavets-Puig, R., Blanc-Mathieu, R., Castro-Mondragon, J. A., Ferenc, K., Kumar, V., et al. (2023). JASPAR 2024: 20th anniversary of the open-access database of transcription factor binding profiles. *Nucleic Acids Res.* 52, D174–D182. doi: 10.1093/nar/gkad1059
